# Impact of an electronic medical record-based appointment order on outpatient cardiology follow-up after hospital discharge

**DOI:** 10.1038/s41746-021-00443-2

**Published:** 2021-05-06

**Authors:** Kartik S. Telukuntla, Chetan P. Huded, Mingyuan Shao, Tim Sobol, Mouin Abdallah, Kathleen Kravitz, Michael Hulseman, Benico Barzilai, Randall C. Starling, Lars G. Svensson, Steven E. Nissen, Umesh N. Khot

**Affiliations:** 1 Heart, Vascular and Thoracic Institute Center for Healthcare Delivery Innovation, Cleveland Clinic, Cleveland, OH USA; 2 Heart, Vascular and Thoracic Institute, Cleveland Clinic, Cleveland, OH USA; 3 Saint Luke’s Mid America Heart Institute, Kansas City, MO USA; 4 C5Research, Department of Cardiovascular Medicine, Cleveland Clinic, Cleveland, OH USA; 5 Heart and Vascular Institute, MedStar Washington Hospital Center, Washington, DC USA; 6 Strata Decision Technology, Chicago, IL USA

**Keywords:** Patient education, Health services

## Abstract

Outpatient follow-up after hospital discharge improves continuity of care and reduces readmissions, but rates of follow-up remain low. It is not known whether electronic medical record (EMR)-based tools improve follow-up. The aim of this study was to determine if an EMR-based order to secure cardiology follow-up appointments at hospital discharge would improve follow-up rates and hospital readmission rates. A pre-post interventional study was conducted and evaluated 39,209 cardiovascular medicine discharges within an academic center between 2012 and 2017. Follow-up rates and readmission rates were compared during 2 years prior to EMR-order implementation (pre-order era 2012–2013, *n* = 12,852) and 4 years after implementation (EMR-order era 2014–2017, *n* = 26,357). The primary endpoint was 90-day cardiovascular follow-up rates within our health system. In the overall cohort, the mean age of patients was 69.3 years [SD 14.7] and 60.7% (*n* = 23,827) were male. In the pre-order era, 90-day follow-up was 56.7 ± 0.4% (7286 of 12,852) and increased to 67.9 ± 0.3% (17,888 of 26,357, *P* < 0.001) in the EMR-order era. The use of the EMR follow-up order was independently associated with increased outpatient follow-up within 90 days after adjusting for patient demographics and payor status (OR 3.28, 95% CI 3.10–3.47, *P* < 0.001). The 30-day readmission rate in the pre-order era was 12.8% (1642 of 12,852) compared with 13.7% (3601 of 26,357, *P* = 0.016) in the EMR-order era. An EMR-based appointment order for follow-up appointment scheduling was associated with increased cardiovascular medicine follow-up, but was not associated with an observed reduction in 30-day readmission rates.

## Introduction

The period after hospitalization has been well documented as a high-risk period during which patients are vulnerable to adverse health outcomes including readmission and death^1^. One-fifth of Medicare patients are readmitted within 30 days of the index hospitalization^2^. Additionally, the median 30-day mortality rate after hospital discharge among Medicare patients is 10–15% for stroke, acute myocardial infarction (AMI), heart failure, and pneumonia^3^.

During this period, timely follow-up after hospital discharge improves transitions in care, reduces readmissions, and may improve mortality^1,2,4^. The follow-up appointment offers an opportunity to provide additional counseling, coordinate recommendations from providers, and ensure proper medication regimen and compliance^5^. Multiple studies have demonstrated that patients who have an early follow-up after discharge have a lower risk of 30-day readmission^2,6^. Additionally, a recent study showed that 30% of AMI patients who were lost to follow-up had higher mortality rates^4,7^.

Though multiple observational studies have shown the benefit of follow-up after hospitalization, rates of follow-up remain low. Only 50% of Medicare patients had a follow-up visit with a physician before readmission^8^. One key factor that has been associated with a higher rate of follow-up is scheduling appointments prior to discharge^9^. However, a Yale-New Haven hospital study suggests that only one-third of patients have scheduled follow-up appointments at the time of discharge^10^. In addition, meaningful interventions have been largely unsuccessful to improve follow-up rates^2,11^.

The Health Information Technology for Economic and Clinical Health Act promoted the rapid adoption of electronic medical records (EMR). As a result, nearly all non-Federal hospitals had certified EMR technologies as of 2015 compared with ~10% in 2008^12^. EMR systems can play a pivotal role in designing comprehensive systems of care to protect our most vulnerable patients. To date, it is unknown whether EMR-based interventions can be leveraged to increase rates of follow-up after discharge. The purpose of this implementation study was to design an EMR-based appointment order to secure cardiovascular medicine follow-up appointments at the time of hospital discharge and to measure the impact on outpatient follow-up rates and 30-day readmission rates.

## Results

### Baseline demographics

The study population included 39,209 patient discharges of which 12,852 discharges were in the pre-order era (2012–2013) and 26,357 discharges were in the EMR-order era (2014–2017). In the overall cohort, 75.7% (*n* = 29,685) of the patients were white, 39.2% were female (*n* = 15,382), and 62.8% (*n* = 24,622) utilized Medicare for health insurance. The average age of patients was 69.3 years [SD 14.7] (Table 1). In the pre-order era, 76.6% (*n* = 9844) of patients were white, 39.3% (*n* = 5055) were female and 60.8% utilized Medicare for health insurance. In the EMR-order era, the patient profile was similar. There was no difference in the population by sex (female sex 39.3% vs. 39.1%, *P* = 0.799) between the two eras. There was a statistically significant difference between race (*P* = 0.007), payor status (*P* < 0.001), and age (*P* < 0.001) in the two groups (pre-order era and EMR-order era).

**Table 1:** Baseline demographics.

Demographics	Overall	Pre-order era	EMR-order era	*P*
	*N* = 39,209	*N* = 12,852	*N* = 26,357	
Age ≥65	65.3%	68.2%	63.9%	<0.001
Female	39.2% (15,382)	39.3% (5055)	39.1% (10,328)	0.799
White	75.7% (29,686)	76.6% (9845)	75.3% (19,841)	0.007
Black	20.8% (8152)	20.2% (2597)	21.1% (5555)	
Other Race	3.5% (1374)	3.2% (413)	3.7% (961)	
Medicare	62.8% (24,623)	60.8% (7813)	63.8% (16,810)	<0.001
Medicaid	8.4% (3299)	5.9% (754)	9.7% (2546)	
Commercial Insurance	25.2% (9889)	28.7% (3867)	23.5% (6202)	
Other Insurance	3.6% (1401)	4.7% (602)	3.03% (799)	

Baseline demographics in the overall, pre-order era, and EMR-order era cohorts.

*EMR electronic medical record.

### Follow-up rates and order utilization

**Fig. 1:**
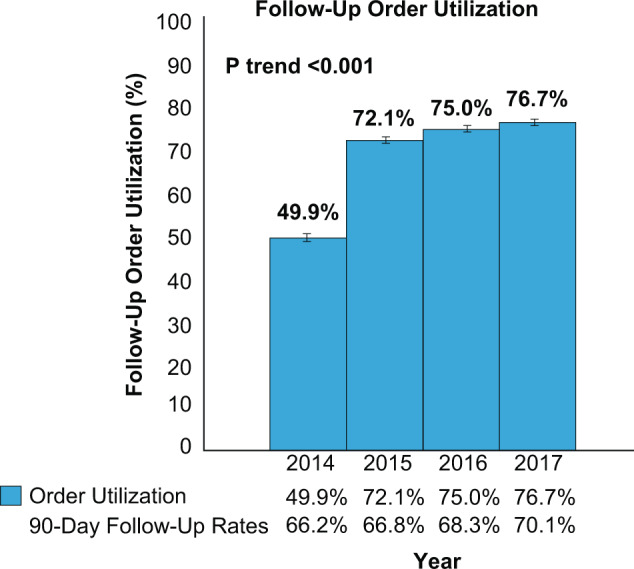
Follow-up order utilization. Within the first year of implementation, 49.9% of discharges utilized the follow-up order showing a strong provider adoption. Our providers continued to use it in an increasing fashion in the subsequent years, demonstrating a sustainable process. (*P* trend 0.001). In addition, the rate of order utilization steadily increased from 49.9% in 2014 to 76.7% in 2017.

In the pre-order era (2012–2013), the 90-day follow-up rate was 56.7 ± 0.4% (7286 of 12,852). In the EMR-order era (2014–2017), the average rate of 90-day follow-up was 67.9 ± 0.3% (17,888 of 26,357, *P* < 0.001) demonstrating a significant increase in follow-up visits after the implementation of the EMR-based appointment order. The average rate of 90-day follow-up was stable at 56.7 ± 0.4% in the pre-order era and then steadily increased from 66.2 ± 0.59% in 2014 to 66.8 ± 0.6% in 2015 to 68.3 ± 0.6% in 2016 to 70.1 ± 0.6% in 2017 (*P* trend < 0.001) during the EMR-order era. During the EMR-order era, utilization of the order by providers increased from 49.9 ± 0.6% of discharges in 2014 to 72.1 ± 0.6% in 2015 to 75.0 ± 0.5% in 2016 to 76.7 ± 0.5% of discharges in 2017 (*P* trend < 0.001) (Fig. 1).

**Fig. 2:**
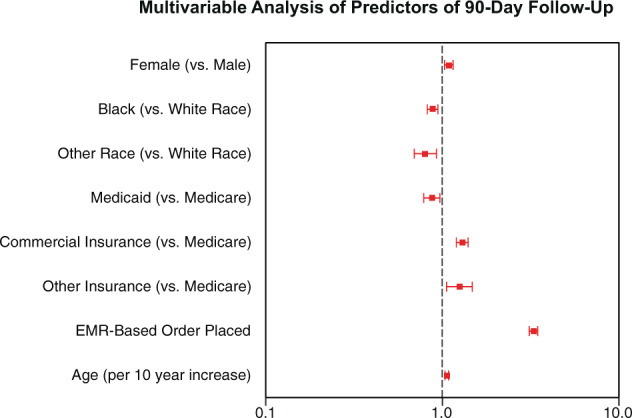
Multivariable analysis of predictors of 90-day follow-up. Key demographic subgroups and their association with 90-day follow-up rates were analyzed using a multivariable logistic regression model. The predictors of 90-day follow-up included female sex, commercial insurance and other insurance (which included self-pay, workers comp), and age (per 10-year increase). Age per 10-year increase OR 1.06 (confidence interval (CI) 1.04–1.08 *P* < 0.001); female: OR 1.09 (CI 1.03–1.15, *P* = 0.003); commercial insurance OR 1.30 (CI 1.20–1.40, *P* < 0.001); other insurance OR 1.25 (CI 1.04–1.08 *p* < 0.009). The strongest predictor of follow-up is the utilization of the EMR-Order (OR 3.28, CI 3.10–3.47, *P* < 0.001). The horizontal axis represents the odds ratio and their 95% confidence intervals on a base-10 log scale. *EMR electronic medical record.

Factors independently associated with 90-day follow-up included female sex (odds ratio [OR] 1.09, 95% confidence interval [CI] 1.03–1.15, *P* = 0.003), commercial insurance (OR 1.30, 95% CI 1.20–1.40, *P* < 0.001, compared to Medicare as reference), other insurance (OR 1.25, 95% CI 1.04–1.08, *P* < 0.009), age (OR 1.06, 95% CI 1.04–1.08, *P* < 0.001, per 10-year increase) and use of the EMR-based appointment order (OR 3.28, 95% CI, CI 3.10–3.47, *P* < 0.001). (Fig. 2).

**Fig. 3:**
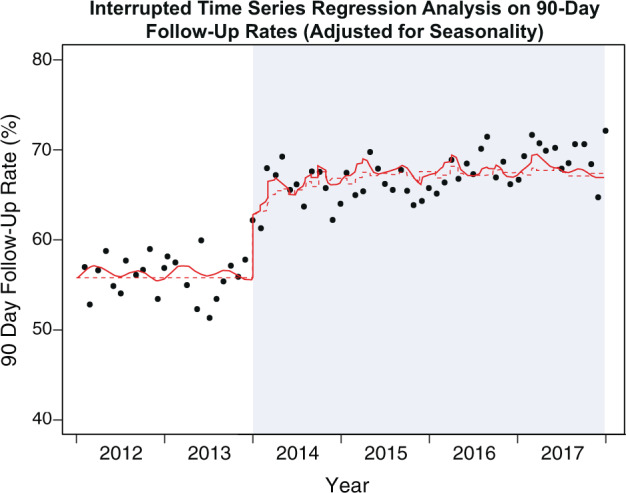
Interrupted time series regression analysis on 90-day follow-up rates (adjusted for seasonality). Interrupted time series of pre-order era and EMR-order era using monthly data based on the Poisson regression model adjusted for seasonality. Solid line: predicted trend based on seasonally adjusted model. Dashed line: de-seasonalized trend.

The unadjusted segmented Poisson regression model for 90-day follow-up over 6 years showed a 14% increase (relative risk (RR) 1.14, 95% CI 1.09–1.19, *P* < 0.001) in the rate of 90-day follow-up after the implementation of the EMR-based appointment order. After adjustment for seasonality through a Fourier term (with no issue on over-dispersion and autocorrelation detected), the impact of the EMR-based appointment order on 90-day follow-up rates was unchanged (RR 1.13, 95% CI 1.10–1.17, *P* < 0.001; Fig. 3). The interrupted time series model for assessing the effect of the EMR-based appointment order utilization on 90-day follow-up showed that the order utilization significantly predicted 90-day follow-up (*P* < 0.001), with an estimated increase of 4.0% in the count of 90-day follow-up (RR 1.0043, 95% CI 1.0038–1.0049) for a 100 count increase in EMR-order use.

The 30-day follow-up rate in the pre-order era was 36.2 ± 0.4% (4648 of 12,852) and increased to 46.8 ± 0.3% (12,343 of 26,357, *P* < 0.001) in the EMR-order era. The 30-day readmission rate in the pre-order era was 12.8 ± 0.29% (1642 of 12,852) compared with 13.7 ± 0.21% (3601 of 26,357, *P* = 0.016) in the EMR-order era. In patients who had a 30-day follow-up visit, the post-follow-up readmission rate up to 30 days post discharge was 6.4% (496 of 7793) in the pre-order era and 7.5% (1363 of 18,107, *P* = 0.001) in the EMR-order era indicating a direct relationship between overall follow-up and overall readmission rates.

### 30-day readmission rates

**Fig. 4:**
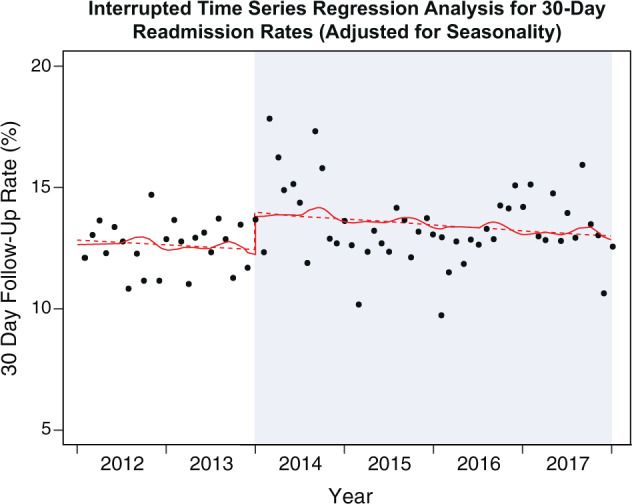
Interrupted time series regression analysis for 30-day readmission rates (adjusted for seasonality). Interrupted time series of pre-order era and EMR-order era using monthly data based on Poisson regression model adjusted for seasonality. Solid line: predicted trend based on seasonally adjusted model. Dashed line: de-seasonalized trend.

The unadjusted segmented Poisson regression model for the 30-day readmission rate over 6 years demonstrated an increase of 13% (RR 1.13, 95% CI 1.02–1.24, *P* = 0.02), indicating an increase in the rate of 30-day readmissions after the implementation of the EMR-based appointment order. After adjustment for seasonality through a Fourier term, the impact of the order on 30-day readmission rates was unchanged (RR 1.13, 95% CI 1.03–1.23, *P* = 0.008; Fig. 4). Figure 4 also demonstrates that monthly readmissions decreased over time in both the pre-order era and EMR-order era.

The bootstrapped mediation analysis showed that the indirect effect of order utilization on readmissions (considering follow-up prior to readmission as a mediator) was estimated to be −0.04 (95% CI: (−0.04, −0.03), *P* < 0.001). The direct effect of order utilization on readmissions (without mediation) was estimated to be 0.05 (95% CI: (0.04, 0.06), *P* < 0.001), demonstrating an inconsistent mediation of follow-up on readmission. The total effect (indirect + direct) of order utilization on monthly readmission was 0.01 (95% CI: (0.01, 0.02), *P* < 0.001). Based on the indirect effect, among patients who followed up within 30 days, the probability of readmission was 3.6% lower than the probability of no readmission when the EMR-based appointment order was utilized.

### Office visits with advanced practice provider

Among advanced practice provider (APP) visits (*N* = 10,500), the overall follow-up rate within 1–7 days was 44.0 ± 0.9% (4623 of 10,500). The 7-day follow-up rate among APP in the pre-order era was 42.6 ± 2.2% (825 of 1937) and increased to 44.4 ± 1.1% (3798 of 8563, *P* = 0.16) in the EMR-order era. In addition, the overall 30-day readmission rate was 14.5 ± 0.7 % (1519 of 10,500) among APP visits. Specifically, it was 14.8 ± 1.6% (287 of 1937) in the pre-order era compared with 14.4 ± 0.7% (1232 of 8563, *P* = 0.63) in the EMR-order era. Regression models adjusting for demographics and insurance information indicate that there was a strong association between the 7-day follow-up and the 30-day readmission in the APP group (Cox regression model: HR and 95% CI, 0.79 (0.72, 0.88), *P* < 0.001; logistic regression model: OR and 95% CI, 0.79 (0.71, 0.89), *P* < 0.001).

## Discussion

This study of 39,209 discharges from the cardiovascular service line at a single large hospital demonstrated that the implementation of an EMR-based appointment order led to a substantial and sustained improvement in the rates of 90-day cardiovascular follow-up after hospital discharge. The order was adopted and utilized by providers in a steadily increasing manner. The EMR-based appointment order was the strongest predictor of 90-day follow-up independent of patient demographics and payor status. This was also observed in the interrupted time series model which demonstrates that order utilization significantly predicted 90-day follow-up. Though the order increased 30-day follow-up rates, paradoxically, it resulted in an increase in 30-day readmission rates.

Observational studies have demonstrated that follow-up after hospital discharge is important, but multiple barriers including logistic, financial, transportation, and socioeconomic inequalities exist in our fragmented health care system making this a challenging task for patients^13^. The simple act of making a follow-up appointment can be extremely difficult for patients. In a survey-based study of ambulatory clinics in multiple US cities, it was found that only 242 of 1065 (23%) telephone calls were able to secure a follow-up appointment within 1 week of an emergency department visit^13^. The EMR-based appointment order streamlined the scheduling process and significantly reduced the logistical barriers to obtain a scheduled appointment ultimately leading to improved follow-up rates.

Patients in a lower socioeconomic status are more likely to be readmitted after the index hospitalization^14^. An analysis of 546,841 index admissions with a principal diagnosis of heart failure in the Nationwide Readmissions Database from 2013 to 2014 showed that patients in the lowest income quartile had a higher readmission rate when compared with those in the highest income quartiles. These findings were confirmed with a multivariable analysis (adjusted OR, 1.11; 95% CI, 1.08–1.13)^14–16^. This study found that a simple EMR-based appointment order improved follow-up rates in all socioeconomic and racial groups. In fact, utilization of the order was the strongest predictor of follow-up when compared with key demographic and socioeconomic factors. Further analysis is needed to determine the order utilization and improvement in follow-up rates stratified by key demographic subgroups. EMR-based interventions can serve as powerful tools to provide benefit to all patients irrespective of socioeconomic status.

The EMR-based order described in this study was implemented in ~25,000 patients over a 4-year period with increasing utilization by providers. The rapid and sustained provider adoption suggests that EMR-based scheduling was favored by providers as it was more efficient, user-friendly, and successful at scheduling follow-up appointments when compared with the traditional model where the primary team has to call and request the appointment. This simple and highly effective intervention can be reproduced and implemented in individual health systems. Due to the promising results of the intervention within the cardiovascular medicine service lines, the EMR-based order has been adopted across all specialties in the entire Cleveland Clinic Health System. Implementation of EMR-based scheduling orders should be explored in all specialties and hospital settings irrespective of the specific EMR that is utilized^17^.

Since Jencks et al. published their landmark study showing that 19.6% of Medicare beneficiaries were readmitted within 30 days, there has been an intense effort to reduce this costly burden including the creation of Hospital Readmissions Reduction Program under the Center for Medicare and Medicaid Services^8^. Because of the observational benefits of follow-up after hospitalization on readmissions, many proposed interventions to improve follow-up have been implemented^18^. During this same period of time, the adoption of EMR systems among US hospitals has been associated with a slight increase in readmissions^19^. This study showed that a simple EMR-based appointment order successfully increased follow-up rates, but also led to an increase in readmission rates. The reason for increased readmissions is likely multifactorial and includes earlier recognition of illness at the time of the office visit, the need to manage acute exacerbations (for example, acute on chronic systolic heart failure), and the generally increased connectivity to the health system resulting in more utilization of care. Furthermore, the follow-up visit was shown in the mediation analysis to be an important contributor to readmissions. Often, sound and meaningful interventions that can improve quality of care may not translate to reductions in readmissions rates as this metric does not fully account for a paradoxical yet competing risk of death^20^. This study highlights the need to reexamine the intense focus on readmissions as a primary metric^7,21^.

This study has several limitations. First, this was a single-center experience with a focus only on the cardiovascular medicine services at the Heart, Vascular, and Thoracic Institute. The pre-order era was a historical control arm. Though this pre-post study has the strength of temporality to suggest the improvement in follow-up rates was impacted by the implementation of the EMR-based appointment order, it does not control for other variables that are also changing at the same time as the implementation of the EMR order^22^. The definition of follow-up in this study was limited to visits with cardiovascular medicine physicians, nurse practitioners, or physician assistants within one health system. Therefore, visits with a non-cardiovascular medicine health professional were not considered in the observed follow-up rates. Additionally, follow-up with providers outside of the health system was not included as data was unavailable. It is possible that if these additional visits were included, the follow-up rates would be higher. The fraction of Medicaid patients was notably different between pre-order (5.9%) and EMR-order (9.7%) eras. The increase in Medicaid patients was likely reflective of the Ohio Medicaid Expansion during the course of the study period. Since 2014, over 1.26 million people enrolled in Ohio Medicaid through the Affordable Care Act-driven expansion^23^. Traditionally, lower rates of follow-up have been observed in Medicaid populations, but this study found that the implementation of the EMR-based appointment order was associated with improved Medicaid follow-up rates in the EMR-order era^14^. The study focused on common demographic variables. Clinical data including primary diagnosis and co-morbidities were not included in order to enhance the generalizability and scalability of this model. All-cause readmissions were included in this study and it is possible some of these admissions were planned readmissions. Impact on mortality was not possible with the available data which could not accurately account for mortality outside of the health system. Lastly, although we strongly believe that the follow-up order was crucial to the findings detailed above, we acknowledge that our implementation study cannot definitively demonstrate a causal relationship.

This study demonstrates that EMR-based interventions are associated with increased follow-up rates after hospital discharge. The available EMR infrastructure in nearly all US hospitals is a highly favorable environment for the widespread adoption of this simple strategy. An EMR-based strategy offers the promise and potential to secure follow-up rates after hospital discharge in an effective and efficient manner.

## Methods

### Study design and participants

The study was conducted at the Heart, Vascular, and Thoracic Institute in the Cleveland Clinic Main Campus. For each patient in the study, the following data were extracted from our EMR system and financial databases: date of index admission, date of index discharge, specific cardiovascular medicine service line, the utilization of the follow-up order, date of follow-up office visit, payor status, demographic information (age, gender, race) and date of readmission. Race was subdivided into three major categories: white, black, other race. Age was divided into two groups: age ≥65 and age <65. Payor status was categorized as follows: Medicare, Medicaid, commercial insurance, other insurance (self-pay, worker’s compensation, unknown insurance). All readmissions, planned and unplanned, for any reason to any hospital within the health system (including the main campus hospital and all regional hospitals in northeast Ohio) within 30 days of the index hospitalization were identified using the institutional billing system. Readmissions to hospitals outside of our health system were not available and not included in the analysis. An internal audit of the institutional readmission tracking system has shown that 78.3% of all readmissions are captured by this method^7^. All 39,209 discharges from the cardiovascular medicine services within the Heart, Vascular, and Thoracic Institute between 2012 and 2017 were included. No patients were excluded.

### Follow-up order process

Between January 1, 2012, and December 31, 2013, the scheduling of follow-up appointments was at the discretion of the primary clinical team. The EMR-based appointment order was created to collect information about the patient’s follow-up needs and was implemented on January 1, 2014. The providers were informed and educated about the new process through emails and announcements at staff meetings. There was no soft roll-out period or best practice advisory fire guiding that was implemented. The order collected the following information: reason for follow-up, request for a specific cardiovascular medicine provider (within 14–30 days), request for whether follow-up with a nurse practitioner or physician assistant was needed between 4 and 7 days. All providers were able to search for the order within Epic, the EMR system used at Cleveland Clinic. Once the order was entered by a provider, it was routed to an Epic pool the HVTI Scheduling Office uses to schedule the follow-up appointment. The follow-up appointment is scheduled in Epic’s standard patient scheduling module called Epic Cadence. The scheduling office was available to execute this process from Monday through Saturday. After the appointment was made, a text page was sent to the ordering provider and the information was automatically entered into the patient discharge instructions (Supplementary Fig. 1). This process is estimated to take less than 2 h from the time of order entry. All orders placed after hours were scheduled on the following business day and notification was sent to the patient if the patient had been discharged.

The primary outcome of this study was 90-day cardiovascular medicine follow-up rates after hospital discharge within the health system presented in a pre-post format and interrupted time series. Office visits with a cardiovascular medicine physician, nurse practitioner, or physician assistant in the health system within 90 days of discharge constituted as follow-up for this study. The secondary outcomes were order utilization over the 4-year period (2014–2017), the trend of 90-day follow-up rates over time (2012–2017), the risk-adjusted association of EMR follow-up with 90-day follow-up rates, and 30-day readmission rates presented in a pre-post format and interrupted time series. The relationship between EMR-order utilization, 30-day follow-up rates, and 30-day readmission rates was studied to understand causality using a bootstrapped mediation analysis.

### Statistical analysis

Demographic information is reported as either mean (one SD) or frequency (percentage). Categorical variables were analyzed with a Pearson chi-square test. The unadjusted rate of 90-day follow-up in the pre-order era vs EMR-order era was compared via a Pearson chi-square test. A Kendall Tau B test of trend was used to assess trends in study order utilization over time and trends in 90-day follow-up rates over time in the EMR-order era. Multivariable logistic regression modeling was used to examine the risk-adjusted association of the EMR follow-up order with 90-day follow-up during the EMR-order era. To match the pre-post interventional study design, a segmented time series was created using the monthly data of 90-day follow-up across the pre-order and EMR-order eras, and a segmented Poisson regression model was fit using the original counts and the offset variable of the age-standardized patient population on the monthly scale^24^. The interrupted time series regression was then extended to use the monthly counts of EMR-order use, for assessing the effect of EMR-order use on 90-day follow-up.

The association between 30-day follow-up rates and 30-day readmission rates was assessed with a Pearson chi-square test. Two analyses were conducted examining the effect of the EMR order on 30-day readmissions: interrupted time series regression model on the monthly 30-day readmission data and a bootstrapped mediation analysis with multivariable logistic regression adjusting for demographics, whereby follow-up status was the mediator. To achieve a casual sequence, when a patient had a follow-up appointment visit that occurred after the day of readmission, the follow-up status was redefined as “no follow-up”.

All analyses were performed with Statistical Package for the Social Sciences (SPSS) software platform (IBM, Armonk, New York) and the open-source R software. *P* < 0.05 indicates statistical significance for all comparisons. This study was approved by the institutional review board at the Cleveland Clinic and a waiver of written informed consent was provided.

### Reporting summary

Further information on research design is available in the Nature Research Reporting Summary linked to this article.

## Supplementary information

Supplementary Information

Reporting Summary

## Data Availability

The data that support the findings of this study are available from the corresponding author upon reasonable request.
