# Supplementary material for: Impact of an electronic medical record-based appointment order on outpatient cardiology follow-up after hospital discharge
Source: NPJ Digit Med. 2021 May 6;4:77. doi: 10.1038/s41746-021-00443-2 (PMC8102598; doi:10.1038/s41746-021-00443-2)

Supplementary Information Figure 1: Follow-Up Order Process

Supplementary Information Figure 1a/b: Follow-Up Appointment Order Screen: Order screen seen by providers. Providers request the division within cardiology, the time frame, any specific provider and provide their contact information. Supplementary Information Figure 1b: Once order is entered, it is routed to a scheduler who makes the appointment. A text page is sent out to the provider to confirm the appointment and the appointment information is also added to the patient’s discharge instructions.

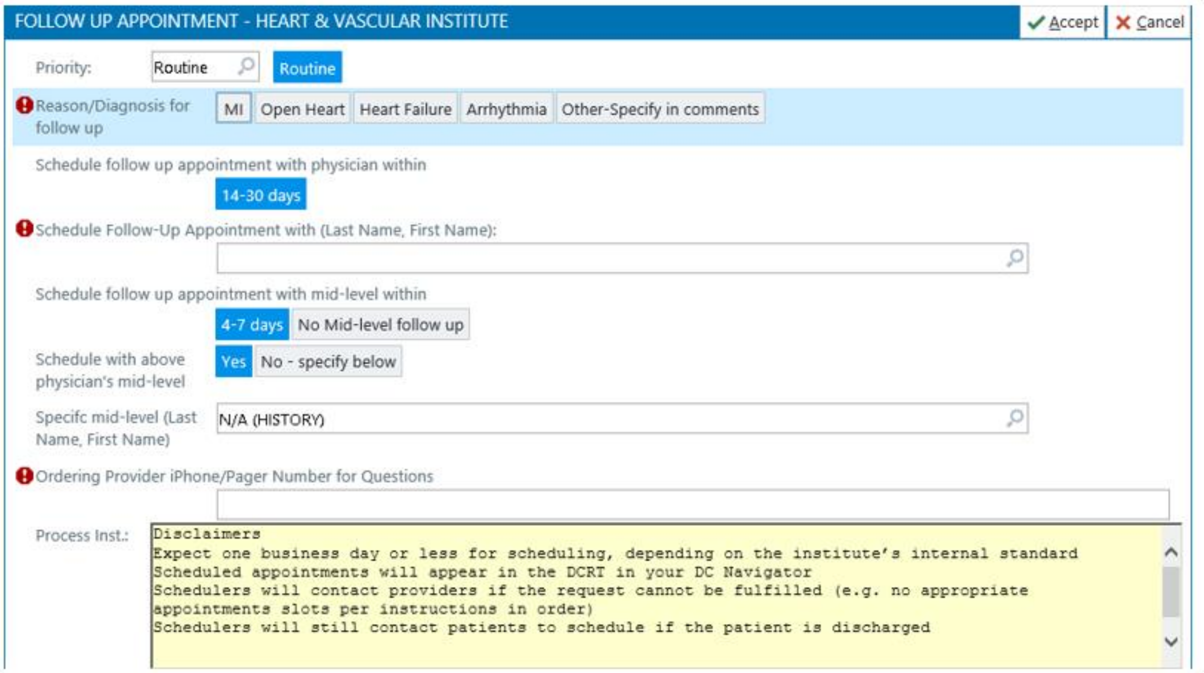

FOLLOW UP APPOINTMENT - HEART & VASCULAR INSTITUTE ✔ Accept ✖ Cancel

Priority: Routine | Routine

Reason/Diagnosis for follow up: MI | Open Heart | Heart Failure | Arrhythmia | Other-Specify in comments

Schedule follow up appointment with physician within: 14-30 days

Schedule Follow-Up Appointment with (Last Name, First Name):

Schedule follow up appointment with mid-level within: 4-7 days | No Mid-level follow up

Schedule with above physician's mid-level: Yes | No - specify below

Specifc mid-level (Last Name, First Name): N/A (HISTORY)

Ordering Provider iPhone/Pager Number for Questions

Process Inst.:
Disclaimers
Expect one business day or less for scheduling, depending on the institute's internal standard
Scheduled appointments will appear in the DCRT in your DC Navigator
Schedulers will contact providers if the request cannot be fulfilled (e.g. no appropriate appointments slots per instructions in order)
Schedulers will still contact patients to schedule if the patient is discharged

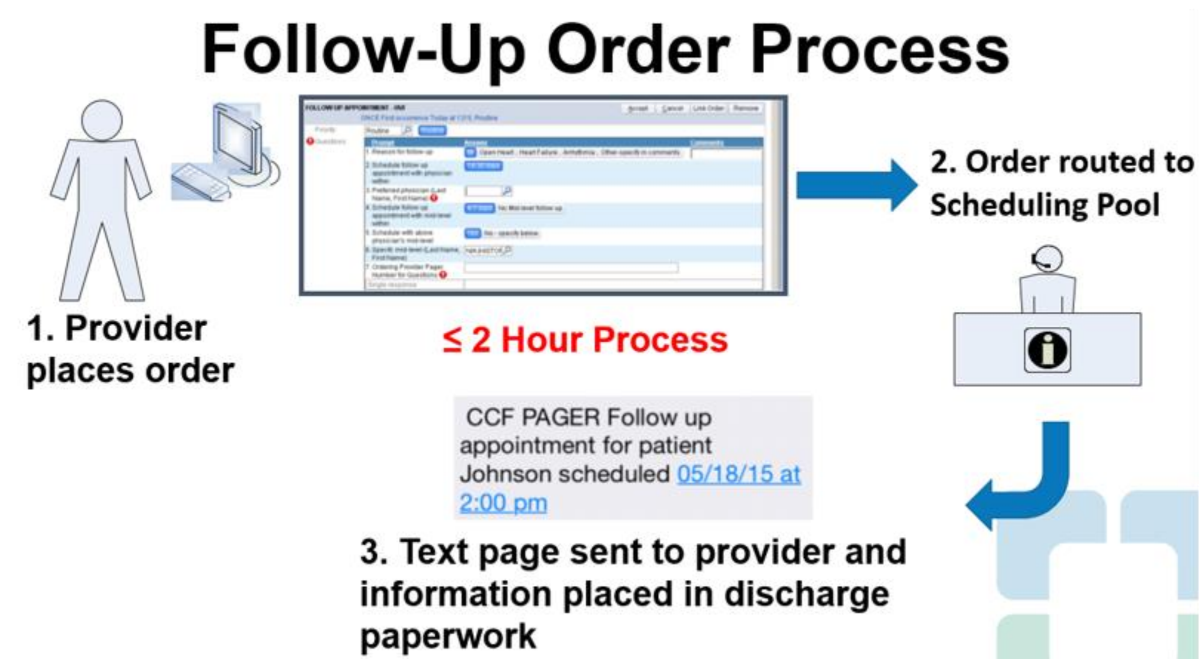

Supplement: Supplementary file 1 — Supplementary Information [file 41746_2021_443_MOESM1_ESM.pdf]
