# Supplementary material for: Impact of an electronic medical record-based appointment order on outpatient cardiology follow-up after hospital discharge
Source: NPJ Digit Med. 2021 May 6;4:77. doi: 10.1038/s41746-021-00443-2 (PMC8102598; doi:10.1038/s41746-021-00443-2)
Supplement: Supplementary file 2 — Reporting Summary [file 41746_2021_443_MOESM2_ESM.pdf]

nature research

Corresponding author(s): Umesh Khot, MD

Last updated by author(s): Jan 9, 2021

Reporting Summary

Nature Research wishes to improve the reproducibility of the work that we publish. This form provides structure for consistency and transparency in reporting. For further information on Nature Research policies, see our Editorial Policies and the Editorial Policy Checklist.

Statistics

For all statistical analyses, confirm that the following items are present in the figure legend, table legend, main text, or Methods section.

n/a	Confirmed	
☐	☒	The exact sample size (n) for each experimental group/condition, given as a discrete number and unit of measurement
☐	☒	A statement on whether measurements were taken from distinct samples or whether the same sample was measured repeatedly
☐	☒	The statistical test(s) used AND whether they are one- or two-sided<br>*Only common tests should be described solely by name; describe more complex techniques in the Methods section.*
☐	☒	A description of all covariates tested
☐	☒	A description of any assumptions or corrections, such as tests of normality and adjustment for multiple comparisons
☐	☒	A full description of the statistical parameters including central tendency (e.g. means) or other basic estimates (e.g. regression coefficient) AND variation (e.g. standard deviation) or associated estimates of uncertainty (e.g. confidence intervals)
☐	☒	For null hypothesis testing, the test statistic (e.g. F, t, r) with confidence intervals, effect sizes, degrees of freedom and P value noted<br>*Give P values as exact values whenever suitable.*
☒	☐	For Bayesian analysis, information on the choice of priors and Markov chain Monte Carlo settings
☒	☐	For hierarchical and complex designs, identification of the appropriate level for tests and full reporting of outcomes
☐	☒	Estimates of effect sizes (e.g. Cohen's d, Pearson's r), indicating how they were calculated

Our web collection on statistics for biologists contains articles on many of the points above.

Software and code

Policy information about availability of computer code

Data collection	All analyses were performed with Statistical Package for the Social Sciences (SPSS) software platform (IBM, Armonk, New York) and the open-source R software.
Data analysis	Demographic information is reported as either mean (one SD) or frequency (percentage). Categorical variables were analyzed with a Pearson chi-square test. The unadjusted rate of 90-day follow-up in the pre-order era vs EMR-order era was compared via a Pearson chi-square test. A Kendall Tau B test of trend was used to assess trends in study order utilization over time and trends in 90-day follow-up rates over time in the EMR-order era. Multivariable logistic regression modeling was used to examine the risk-adjusted association of the EMR follow-up order with 90-day follow-up during the EMR-order era. To match the pre-post interventional study design, a segmented time series was created using the monthly data of 90-day follow-up across the pre-order and EMR-order eras, and a segmented Poisson regression model was fit using the original counts and the offset variable of the age-standardized patient population on the monthly scale13. The interrupted time series regression was then extended to use the monthly counts of EMR order use, for assessing the effect of EMR order use on 90-day follow-up. The association between 30-day follow-up rates and 30-day readmission rates was assessed with a Pearson chi square test. Two analyses were conducted examining the effect of the EMR order on 30-day readmissions: interrupted time series regression model on the monthly 30-day readmission data and a bootstrapped mediation analysis with multivariable logistic regression adjusting for demographics, whereby follow-up status was the mediator. To achieve a casual sequence, when a patient had a follow-up appointment visit that occurred after the day of readmission, the follow-up status was redefined as "no follow-up".

For manuscripts utilizing custom algorithms or software that are central to the research but not yet described in published literature, software must be made available to editors and reviewers. We strongly encourage code deposition in a community repository (e.g. GitHub). See the Nature Research guidelines for submitting code & software for further information.

Data

Policy information about availability of data

All manuscripts must include a data availability statement. This statement should provide the following information, where applicable:

- Accession codes, unique identifiers, or web links for publicly available datasets
- A list of figures that have associated raw data
- A description of any restrictions on data availability

Provide your data availability statement here.

Field-specific reporting

Please select the one below that is the best fit for your research. If you are not sure, read the appropriate sections before making your selection.

☐ Life sciences ☒ Behavioural & social sciences ☐ Ecological, evolutionary & environmental sciences

For a reference copy of the document with all sections, see nature.com/documents/nr-reporting-summary-flat.pdf

Behavioural & social sciences study design

All studies must disclose on these points even when the disclosure is negative.

Study description	The aim of this study was to determine if an EMR-based order to secure cardiology follow-up appointments at the time of hospital discharge would improve follow-up rates and hospital readmission rates.
Research sample	39,209 discharges from the cardiovascular medicine services within an academic center between 2012 and 2017
Sampling strategy	no sampling strategy, we used all discharges from the CV medicine line from 2012-2017
Data collection	For each patient in the study, the following data was extracted from our EMR system and financial databases: date of index admission, date of index discharge, specific cardiovascular medicine service line, the utilization of the follow-up order, date of follow-up office visit, payor status, demographic information (age, gender, race) and date of readmission.
Timing	2012-2017
Data exclusions	none
Non-participation	none
Randomization	no randomization

Reporting for specific materials, systems and methods

We require information from authors about some types of materials, experimental systems and methods used in many studies. Here, indicate whether each material, system or method listed is relevant to your study. If you are not sure if a list item applies to your research, read the appropriate section before selecting a response.

Materials & experimental systems

n/a	Involved in the study
☒	☐ Antibodies
☒	☐ Eukaryotic cell lines
☒	☐ Palaeontology and archaeology
☒	☐ Animals and other organisms
☒	☐ Human research participants
☒	☐ Clinical data
☒	☐ Dual use research of concern

Methods

n/a	Involved in the study
☒	☐ ChIP-seq
☒	☐ Flow cytometry
☒	☐ MRI-based neuroimaging
